# Ultrathin, Lightweight, and Flexible CNT Buckypaper Enhanced Using MXenes for Electromagnetic Interference Shielding

**DOI:** 10.1007/s40820-021-00597-4

**Published:** 2021-02-09

**Authors:** Rongliang Yang, Xuchun Gui, Li Yao, Qingmei Hu, Leilei Yang, Hao Zhang, Yongtao Yao, Hui Mei, Zikang Tang

**Affiliations:** 1 State Key Laboratory of Optoelectronic Materials and Technologies, School of Electronics and Information Technology, Sun Yat-Sen University, Guangzhou, 510275 People’s Republic of China; 2 Science and Technology on Thermostructural Composite Materials Laboratory, School of Materials Science and Engineering, Northwestern Polytechnical University, Xi’an, 710072 Shaanxi People’s Republic of China; 3 Instrumental Analysis and Research Center (IARC), Sun Yat-Sen University, Guangzhou, 510275 People’s Republic of China; 4 National Key Laboratory of Science and Technology on Advanced Composites in Special Environments, Harbin Institute of Technology, Harbin, 150080 People’s Republic of China; 5 Institute of Applied Physics and Materials Engineering, University of Macau, Taipa, 999078 Macau People’s Republic of China

**Keywords:** Carbon nanotube, MXene, Buckypaper, Electromagnetic interference shielding

## Abstract

Lightweight, flexibility, and low thickness are urgent requirements for next-generation high-performance electromagnetic interference (EMI) shielding materials for catering to the demand for smart and wearable electronic devices. Although several efforts have focused on constructing porous and flexible conductive films or aerogels, few studies have achieved a balance in terms of density, thickness, flexibility, and EMI shielding effectiveness (SE). Herein, an ultrathin, lightweight, and flexible carbon nanotube (CNT) buckypaper enhanced using MXenes (Ti_3_C_2_T_x_) for high-performance EMI shielding is synthesized through a facile electrophoretic deposition process. The obtained Ti_3_C_2_T_x_@CNT hybrid buckypaper exhibits an outstanding EMI SE of 60.5 dB in the X-band at 100 μm. The hybrid buckypaper with an MXene content of 49.4 wt% exhibits an EMI SE of 50.4 dB in the X-band with a thickness of only 15 μm, which is 105% higher than that of pristine CNT buckypaper. Furthermore, an average specific SE value of 5.7 × 10^4^ dB cm^2^ g^−1^ is exhibited in the 5-μm hybrid buckypaper. Thus, this assembly process proves promising for the construction of ultrathin, flexible, and high-performance EMI shielding films for application in electronic devices and wireless communications.

**Supplementary Information:**

The online version contains supplementary material available at 10.1007/s40820-021-00597-4.

## Highlights

- Ultrathin, lightweight, and flexible carbon nanotube buckypaper enhanced using MXenes (Ti_3_C_2_T_x_) for high-performance electromagnetic interference shielding is synthesized through facile electrophoretic deposition.
- The obtained Ti_3_C_2_T_x_@CNT hybrid buckypaper demonstrates outstanding EMI shielding effectiveness of 60.5 dB in the X-band at 100 μm and a specific SE value of 5.7 × 10^4^ dB cm^2^ g^−1^ at 5 μm.

**Supplementary Information:**

The online version contains supplementary material available at 10.1007/s40820-021-00597-4.

## Graphical abstract

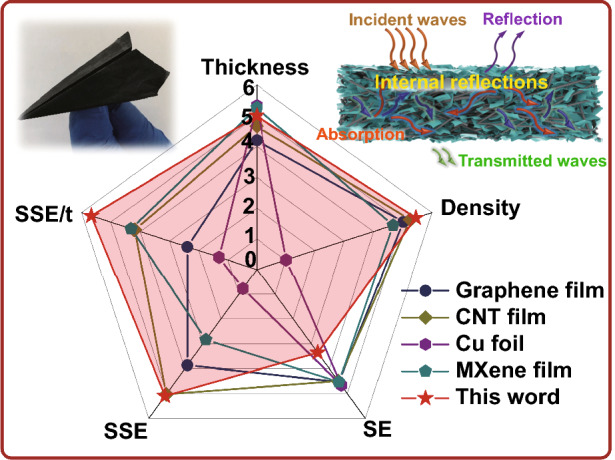

## Introduction

In the rapidly developing age of electronics, because the increasing amount of electromagnetic radiation pollution is damaging the stability of electronic equipment and even human health, high-performance electromagnetic interference (EMI) shielding materials are in great demand. Instead of traditional metal-based materials, next-generation EMI shielding materials are required and must be flexible, lightweight, and highly efficient. Carbon-based nanomaterials, including cellulose nanofibers [1, 2], carbon nanotubes (CNTs) [3–5], and graphene [6, 7], are widely considered as promising, flexible EMI shielding materials because of their low density, high conductivity, and high processability. For instance, the CNT sponge/epoxy composite exhibits an EMI shielding effectiveness (SE) of 33 dB in the X-band at 2 mm [3]. In addition, graphene/polydimethylsiloxane (PDMS) foam has shown a high EMI SE of 30 dB in the X-band at a thickness of 1.0 mm [6]. To further decrease the density and improve the specific SE (SSE), CNTs or graphene have been directly used to construct shielding films with porous network structures [8–11]. For example, a graphene-based aerogel with a density of only 24.5 mg cm^−3^ showed a high EMI SE of 83 dB in the X-band at a thickness of 2.0 mm [10]. A free-standing CNT sponge with a density of 10.0 mg cm^−3^ exhibited a satisfactory EMI SE of 54.8 dB and an SSE of 5480 dB cm^3^ g^−1^ at a thickness of 1.8 mm [12]. Nevertheless, because absorption is the dominant EMI shielding mechanism in these carbon-based materials [4, 12], the shielding performance relies on the thickness of the samples. Almost all carbon-based nanomaterials are required to have a high thickness to achieve a high SE. The construction of a flexible high-performance EMI shielding material with a low thickness remains a challenge.

In the case of a limited thickness (resulting in limited absorption), improving the reflection is one of the most effective strategies to enhance the EMI performance. Many efforts are being focused on improving the conductivity and optimizing shielding material microstructures. All types of metallic nanoparticles [13], nanowires [14, 15], and nanosheets have been used to combine with carbon nanomaterials to achieve a high conductivity and thereby a high shielding performance with limited thickness. In particular, MXene nanosheets have attracted significant interest for EMI shielding owing to their metallic conductivity and unique two-dimensional (2D) structure [16–19]. Although densely packed MXene can exhibit a high EMI SE at a low thickness [20–25], dense MXene films may not satisfy the requirements of flexibility and low density owing to the weak interlayer interactions between MXene nanosheets [26, 27]. Combining carbon nanomaterials with MXene is a promising strategy for balancing the mechanical properties and SE [1, 9, 28–30]. Recently, A reported flexible CNT/MXene/cellulose nanofibril hybrid film exhibited a high conductivity of 2506.6 S m^−1^ and an SE of 38.4 dB in the X-band at a thickness of 38 μm [31]. Combined with MXene sheets, a highly dense graphene oxide film with a layered structure has shown an excellent SE of 50.2 dB at a low thickness of 7 μm [32]. Nevertheless, because most of these studies have combined carbon materials with MXene via the mixing of their fragments through dispersion and obtained a shielding film using a spraying or filtration process, there are still several disadvantages to such films. First, although carbon materials and MXene nanosheets can be well dispersed in a solution, during the spraying or filtration process, the MXene nanosheets tend to self-stack when the solvent decreases. Although some reports have employed a layer-by-layer assembly method to avoid aggregation problems [26, 33–35], this is a complicated process. Second, the filtration process for 2D materials is time-consuming and unsuitable for large-scale production. Finally, the nanosheets are usually connected through a hydrogen bond or Van der Waals forces. These connections may not be sufficiently stable to withstand harsh situations. Thus, a more efficient assembly process for a carbon-based/MXene EMI shielding film should be developed.

Here, a simple assembly process is developed for fabricating a flexible MXene-enhanced CNT buckypaper with a satisfactory EMI SE within a small thickness. Pristine CNT buckypaper consisting of a porous and continuous CNT network is obtained via a typical chemical vapor deposition (CVD) process. After a simple rolling process, the compressed CNT buckypaper is observed to show a high SE of 49.8 dB in the X-band at a thickness of 100 μm, which is higher than that of most of the reported carbon materials. After a facile electrophoretic deposition process, the MXene (Ti_3_C_2_T_x_) nanoflakes infiltrate into the CNT network and wrap around the CNT skeleton. The homogeneously distributed Ti_3_C_2_T_x_ nanoflakes are tightly connected to the CNTs, resulting in a higher shielding performance than that of the randomly stacked Ti_3_C_2_T_x_/CNT composite. The obtained Ti_3_C_2_T_x_@CNT hybrid buckypaper exhibits an outstanding SE of 60.5 dB in the X-band at a thickness of 100 μm. Moreover, the shielding performance of the hybrid buckypaper can be easily adjusted by changing the thickness and MXene content. The ultrathin 15-μm hybrid buckypaper with 49.4 wt% Ti_3_C_2_T_x_ exhibits an SE of 50.4 dB in the X-band, which is 105% higher than that of CNT buckypaper. In addition, an average specific SE (SSE/t) value of 56,945.8 dB cm^2^ g^−1^ is exhibited in the 5-μm hybrid buckypaper. Thus, the novel hybrid film is a promising high-performance EMI shielding material.

## Experimental Section

### Synthesis of CNT Buckypaper

A traditional CVD method was used to synthesize the free-standing and flexible CNT buckypaper, for which a solution of ferrocene and dichlorobenzene was used as a carbon source. The flow rate of the carrying gas was set as 2000/300 sccm of Ar/H_2_, and the growth temperature was set as 860 °C. Through setting the growth time, CNT buckypapers with a series of thicknesses from 0.1 to 2.0 mm were obtained. Then, a simple rolling process was used to densify the CNT buckypapers, the densities of which increased from ~ 10 to ~ 500 mg cm^−3^. Accordingly, the thickness decreased to 5–100 μm.

### Synthesis of Ti_3_C_2_T_x_ Nanosheets

The typical selective etching process was used to obtain MXene (Ti_3_C_2_T_x_) nanosheets from the MAX phase precursor (Ti_3_AlC_2_). In a polytetrafluoroethylene container, 2 g of LiF powder was added to 20 mL of 9 M HCl solution. Subsequently, at a constant temperature of 35 °C, 1 g of Ti_3_AlC_2_ powder was slowly added to the container, followed by continuous stirring. After a 24-h reaction, the reactant was centrifugally washed several times, until the pH > 6. The obtained sediment was collected and dispersed in DI water again with 1-h sonication in an ice bath and flowing argon atmosphere. Finally, after dilution and centrifugal separation at 8000 rpm several times, the dark green Ti_3_C_2_T_x_ nanosheet supernatant was collected.

### Synthesis of Ti_3_C_2_T_x_@CNT Hybrid Buckypaper

The Ti_3_C_2_T_x_@CNT hybrid buckypaper was prepared through a simple electrophoretic deposition process. As the working electrode, the as-grown porous CNT buckypapers with different thicknesses (0.1–2.0 mm) were cut into regular squares, while Pt was used as the counter electrode, and 1.0 mg mL^−1^ Ti_3_C_2_T_x_ aqueous dispersion was used as the electrolyte. With a controlled applied constant voltage (10 V) and reaction time (10–120 min), a series of Ti_3_C_2_T_x_@CNT hybrids were obtained, after which they were vacuum dried at 50 °C for 12 h. Finally, the hybrids were densified through the same rolling process to obtain the Ti_3_C_2_T_x_@CNT hybrid buckypapers, the thicknesses of which decreased to 5–100 μm.

### Characterization

The microstructures and morphologies of the Ti_3_C_2_T_x_ nanosheets, CNT buckypaper, and Ti_3_C_2_T_x_@CNTs hybrid buckypaper were characterized using scanning electron microscopy (SEM, Hitachi, S-4800) and transmission electron microscopy (TEM, Tecnai, F30). The composition ratio in the hybrid buckypaper was tested through thermogravimetric analysis (TGA, Netzsch, TG209 F1). The crystal structures, ingredient information, and chemical bonds in the samples were measured using X-ray photoelectron spectroscopy (XPS, Thermo Scientific, ESCALAB 250Xi), X-ray powder diffraction (XRD, Empyrean), and the Raman spectra (HORIBA, LabRAM HR). The EMI SE was determined using a vector network analyzer (Agilent Technologies, N5234A) within the frequency range of 8–12 GHz. The electrical conductivity of the samples was measured using a Keithley 2400.

## Results and Discussion

### Shielding Performance of CNT Buckypaper

**Fig. 1:**
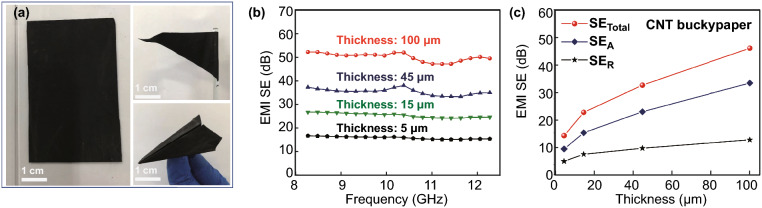
EMI performance of CNT buckypaper. **a** Digital image of flat CNT buckypaper, rolled-up flag, and folded paper plane. **b** EMI SE of CNT buckypapers with different thickness in X-band region. **c** Comparison of average SE_Total_, SE_A_, and SE_R_ versus thickness of CNT buckypapers

Free-standing, flexible CNT buckypapers consisting of porous and continuous CNT networks were synthesized through a well-controlled CVD process. A simple rolling process was used to densify the CNT buckypapers (Fig. S1), in which process the densities of the CNT buckypaper increased from ~ 10 to ~ 500 mg cm^−3^, and the thickness decreased from 0.1–2.0 mm to 5–100 μm. The interconnected CNTs ensured that the CNT network endured a complex change in shape. A high structural stability and flexibility are demonstrated in the densified films. The films can be folded into a “paper plane” without undergoing any breaks or tears, as shown in Fig. 1a. The continuous CNT network also leads to satisfactory electronic transmission performance. A high electrical conductivity of ~ 7000 S m^−1^ was observed in the densified films. The highly conductive network endows CNT buckypaper with satisfactory EMI shielding performance. The total SE (SE_Total_) shows an evident increase with the added film thickness (Fig. 1b). Over the entire X-band, the average SE_Total_ of the 5-μm CNT buckypaper is 15.4 dB, whereas that of the 100-μm CNT buckypaper increases to a high value of 49.8 dB. To further discuss the EMI shielding mechanism in the samples, the EMI reflection (SE_R_) and EMI absorption (SE_A_) over the X-band have also been investigated. As shown in Fig. S2, all samples with different thicknesses exhibit strong microwave absorption in the X-band. With an increase in thickness, the average SE_A_ shows a distinctly rising trend, whereas the SE_R_ grows quite slowly (Fig. 1c). These trends demonstrate an absorption-dominant mechanism in CNT buckypaper, and the reflection loss needs to be improved. The high absorption ratio may be attributed to the dense and porous network structure. SE_Total_ data of more CNT buckypaper with different thicknesses are shown in Fig. S2e, which further indicates the adjustability in the shielding performance of the CNT buckypaper. The free-standing CNT buckypaper synthesized in this work shows a high EMI shielding performance among all carbon nanomaterials. However, there is still plenty of room for improvement.

### Synthesis and Characterization of Ti_3_C_2_T_x_@CNT Hybrid Buckypaper

**Fig. 2:**
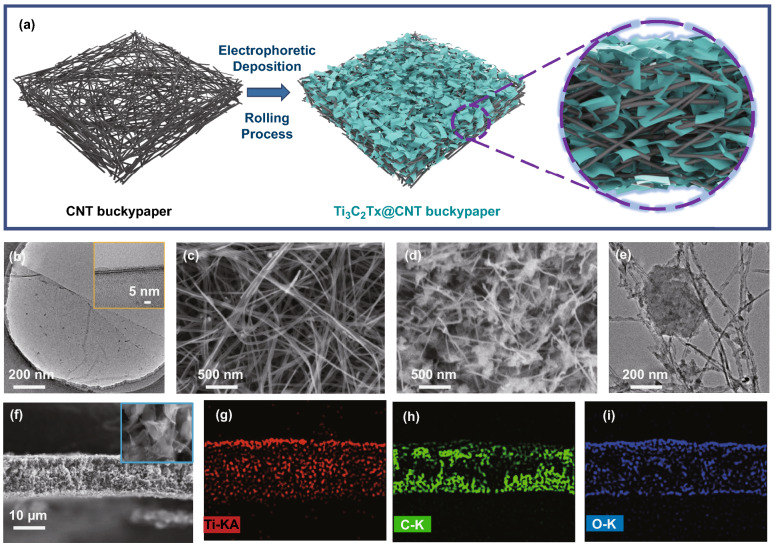
Morphology of Ti_3_C_2_T_x_@CNT hybrid buckypaper. **a** Schematic illustration of electrophoretic deposition process for fabrication of Ti_3_C_2_T_x_@CNT hybrid buckypaper. **b** TEM image of Ti_3_C_2_T_x_ nanosheet (the inset shows the corresponding high-resolution TEM image). SEM image for interior of **c** CNT buckypaper and **d** Ti_3_C_2_T_x_@CNT hybrid buckypaper. **e** TEM image of Ti_3_C_2_T_x_@CNT hybrid. **f** Cross-sectional SEM image of hybrid buckypaper (the inset shows the local details at high resolution). The corresponding EDS mapping images of **g** Ti, **h** C, and **i** O

MXene (Ti_3_C_2_T_x_) nanosheets were used to enhance the CNT buckypaper for EMI shielding. As illustrated in Fig. 2a, a simple and novel electrophoretic deposition process was used to prepare the Ti_3_C_2_T_x_@CNT hybrid buckypaper. During this process, the free-standing and porous CNT buckypaper was used as the working electrode and supporter, whereas Ti_3_C_2_T_x_ nanosheets dispersed in water were employed as fillers. As the fillers, Ti_3_C_2_T_x_ nanosheets were obtained by selectively etching from the MAX phase precursor (Ti_3_AlC_2_). The exfoliated Ti_3_C_2_T_x_ nanosheets show a structure with a few layers, ~ 5 nm in thickness and a mean size of several hundred nanometers to several micrometers (Figs. 2b and S3a). The crystal structure of the Ti_3_C_2_T_x_ nanosheet was further investigated through XRD patterns (Fig. S3b). When compared with that of Ti_3_AlC_2_, the characteristic peak (002) of Ti_3_C_2_T_x_ clearly shifts from 2*θ* = 9.5° to 2*θ* = 5.7°, revealing the removal of Al layers and, thereby, enlargement of the c-lattice parameter in the Ti_3_C_2_T_x_ [19, 35]. Moreover, after exfoliation, the (104) peak of Ti_3_C_2_T_x_ at 2*θ* = 39° significantly decreases, which also indicates the successful selective etching of Al atoms and, thereby, decrease in the structural order. The exfoliated Ti_3_C_2_T_x_ nanosheets were further selected through centrifugal separation at 8000 rpm. The selected Ti_3_C_2_T_x_ nanosheets with smaller sizes were dispersed as 1.0 mg mL^−1^ aqueous dispersion (Fig. S4b), which showed a zeta potential of – 26.52 mV (Fig. S4c). The size distribution results demonstrate that the diameters of the Ti_3_C_2_T_x_ nanosheets are concentrated around 288 nm, which proves suitable for infiltration into the CNT network.

As the working electrode, the CNT buckypapers were full of pores, which allowed for the effective infiltration of the Ti_3_C_2_T_x_ nanoflakes (Fig. 2c). During the electrophoretic deposition process (Fig. S4a), when a certain voltage (10 V) was applied, the Ti_3_C_2_T_x_ nanosheets with negative charges in dispersion were forced to move toward the CNT buckypaper and finally infiltrate the carbon nanotube network. The size distributions of Ti_3_C_2_T_x_ nanoflakes around the two electrodes show that small-sized nanoflakes were firstly gathered at the CNT electrode (Fig. S4d). As the deposition process continues, the deposition process becomes stable. Moreover, under the applied potential, both the carbon nanotubes and Ti_3_C_2_T_x_ nanosheets can be slightly etched, as proved in many reports [36–38]. The slightly unzipped and etched nanotube network provides a larger space for the infiltration of flakes, whereas the Ti_3_C_2_T_x_ nanosheets can also be fragmented into a smaller size. After electrophoretic deposition, the CNT buckypaper was covered with a uniform Ti_3_C_2_T_x_ layer and maintained its flexibility (Fig. S5a-c). Metalloid coatings can effectively enhance the reflection loss of electromagnetic waves. Meanwhile, the internal morphology of the Ti_3_C_2_T_x_@CNT hybrid buckypaper clearly shows homogeneously distributed Ti_3_C_2_T_x_ inside the CNT network (Fig. 2d). The sizes of the inserted Ti_3_C_2_T_x_ nanoflakes are approximately tens to hundreds of nanometers, which can be further demonstrated in the TEM image (Fig. 2e). Cross-sectional SEM images and the related energy-dispersive spectrometry (EDS) mapping images further indicate the uniform distribution of Ti_3_C_2_T_x_ nanoflakes in a hybrid buckypaper (Fig. 2f–i). The inserted Ti_3_C_2_T_x_ nanoflakes can uniformly fill the entire CNT buckypaper (Fig. 2g). More details of the cross-sectional SEM image of the Ti_3_C_2_T_x_@CNT hybrid buckypaper are shown in Fig. S5d-i. Both the inserted Ti_3_C_2_T_x_ nanoflakes and the CNT network formed a large number of micro-fissures. These separated nanoflakes and the formed micro-fissures greatly enhanced the internal multiple reflection and absorption of the film for electromagnetic waves. Moreover, the synthesized hybrid buckypapers were also densified via the same rolling process, in which Ti_3_C_2_T_x_@CNT hybrid buckypapers with thicknesses of 5–100 μm were obtained.

**Fig. 3:**
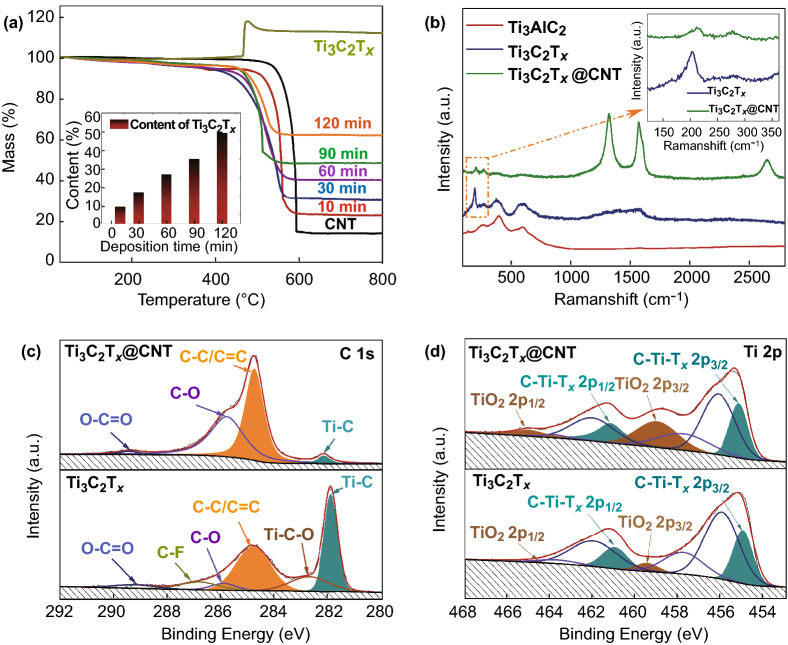
Structural characterization of Ti_3_C_2_T_x_@CNT hybrid buckypaper. **a** TG curves of Ti_3_C_2_T_x_, CNT buckypaper, and Ti_3_C_2_T_x_@CNT hybrid buckypaper with different deposition time (the inset is the calculated content of Ti_3_C_2_T_x_ in hybrid buckypapers with a thickness of 15 μm). **b** Raman spectra of Ti_3_AlC_2_, Ti_3_C_2_T_x_, and Ti_3_C_2_T_x_@CNT hybrid buckypaper (the inset is the partially enlarged spectra). High-resolution XPS spectra of **c** C 1*s* and **d** Ti 2*p* for Ti_3_C_2_T_x_ and Ti_3_C_2_T_x_@CNT hybrid buckypaper

The content of Ti_3_C_2_T_x_ nanoflakes in the hybrid buckypaper can be controlled by regulating the deposition time and applied voltage. In this work, with an applied voltage of 10 V, the content of Ti_3_C_2_T_x_ can be effectively increased by extending the deposition time. For instance, for the 15-μm samples, the content of Ti_3_C_2_T_x_ in the hybrid buckypaper is 9.2 wt% and 49.4 wt% after 10- and 120-min deposition, respectively, which can be calculated using the TG curves in air (Fig. 3a). Raman spectra further confirm the successful synthesis of the Ti_3_C_2_T_x_ nanoflakes and Ti_3_C_2_T_x_@CNT hybrid buckypaper (Fig. 3b). The characteristic peak of Ti atoms at 199 cm^−1^ (corresponding to the out-of-plane vibrations) and that at 283 cm^−1^ (corresponding to the in-plane modes) can be found in both the Ti_3_C_2_T_x_ and Ti_3_C_2_T_x_@CNT curves. Meanwhile, for the Ti_3_C_2_T_x_@CNTs, the two intense peaks at 1328 cm^−1^ (D band) and 1577 cm^−1^ (G band) can be related to the carbon nanotubes. These typical characteristic peaks indicate a good combination of Ti_3_C_2_T_x_ and CNTs. Detailed elemental chemical states in the Ti_3_C_2_T_x_@CNT hybrid buckypaper were further investigated using XPS (Fig. S3c). Both the Ti_3_C_2_T_x_ and Ti_3_C_2_T_x_@CNT hybrid buckypapers show distinct signals of F and O, which are attributed to surface terminations such as –F, –OH, and –O [39]. Among them, the slightly increased O 1*s* signal in the Ti_3_C_2_T_x_@CNT hybrid originates from the minor oxidation during the electrophoretic deposition process. To confirm the chemical bonding in the Ti_3_C_2_T_x_@CNT hybrid, high-resolution C 1*s* and Ti 2*p* spectra were further studied (Fig. 3c, d). In the C 1*s* spectra, the sharply increased C–C/C=C signal (284.8 eV) in the Ti_3_C_2_T_x_@CNT hybrid originates from the combination of Ti_3_C_2_T_x_ with carbon nanotubes. Meanwhile, in the Ti 2*p* spectra, the Ti–C doublet peak (455.0 eV and 461.0 eV) remains almost constant in the Ti_3_C_2_T_x_@CNT hybrid, whereas the TiO_2_ doublet peak (459.5 eV and 465.5 eV) increases slightly. The added oxygen signal in the Ti_3_C_2_T_x_@CNT hybrid is mainly attributed to oxidized carbon.

### Shielding Performance of Ti_3_C_2_T_x_@CNT Hybrid Buckypaper

**Fig. 4:**
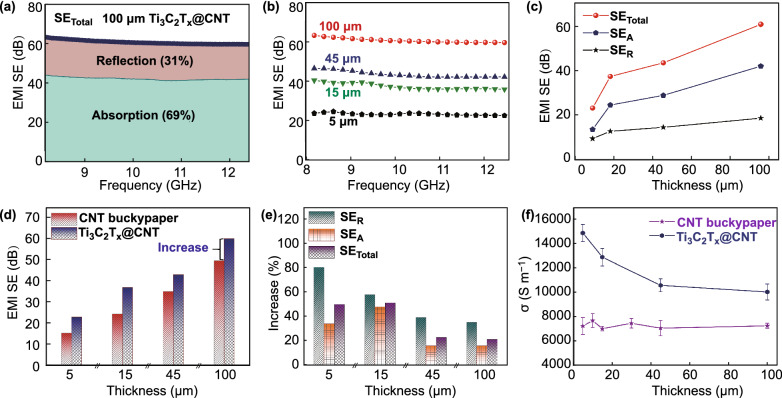
EMI performance of Ti_3_C_2_T_x_@CNT hybrid buckypaper with different thickness. **a** EMI SE of 100-μm hybrid buckypaper (the SE_Total_ includes the reflection and absorption parts). **b** EMI SE of the hybrid buckypapers with different thickness. **c** Average SE_Total_, SE_A_, and SE_R_ versus thickness for Ti_3_C_2_T_x_@CNT hybrid buckypapers. **d** Comparison of average SE_Total_ versus thickness between CNT buckypapers and the hybrid buckypapers. **e** The increasing ratio of average SE_Total_, SE_A_, and SE_R_ versus thickness in Ti_3_C_2_T_x_@CNT hybrid buckypapers (compared with the pristine CNT buckypaper). **f** Comparison of electrical conductivities versus thickness between CNT buckypaper and the hybrid buckypapers

To investigate the influence of filling Ti_3_C_2_T_x_ on EMI SE, Ti_3_C_2_T_x_@CNT hybrid buckypapers with various thicknesses were synthesized through a fixed electrophoretic deposition time (30 min). After the deposition process, all hybrids remained at almost the same thickness as that of the pristine CNT buckypapers. After the rolling process, the prepared 100-μm hybrid buckypaper exhibited a high average SE_Total_ of 60.5 dB within the X-band region (Fig. 4a). Although absorption was still the main shielding mechanism in hybrid buckypapers, its reflection loss ratio increased to 31%. Detailed EMI SE curves of hybrid buckypapers with various thicknesses within the X-band are shown in Figs. 4b and S6a–d. The SE_Total_, SE_R_, and SE_A_ values in the hybrid buckypapers all show increasing trends with the added thickness, which are similar to those of the pristine CNT buckypapers (Fig. 4c). The enhanced reflection may be attributed to the MXene coating and increase the electrical conductivity, whereas the enhanced absorption may contribute to interface polarization and internal reflection.

A comparison of the SE between the Ti_3_C_2_T_x_@CNT hybrid buckypapers and pristine CNT buckypapers is shown in Figs. 4d and S6. An approximate increase in the SE values is observed in samples with different thicknesses. The 15-μm hybrid buckypaper presents a 12.6-dB increase in SE_Total_, whereas the 100-μm sample shows a 10.6-dB increment. Thus, for the hybrid buckypapers with various thicknesses, the electrophoretic deposition processes with fixed deposition time contributed to efficient and stable enhancements in the shielding performance. Nevertheless, because the CNT buckypapers with small thicknesses show a significantly lower SE, a higher increasing ratio appears in thinner samples (Fig. 4e). For instance, when compared with the pristine CNT buckypaper, the 5-μm hybrid buckypaper presents an increasing ratio of 80.8% on SE_R_. Meanwhile, for the 100-μm hybrid buckypaper, the increasing ratio of SE_R_ is only 35.5%. It should be noted that when compared with the 15-μm hybrid buckypaper, the 5-μm sample shows a higher increasing ratio on SE_R_ but a lower ratio on SE_A_ and SE_Total_. This indicates that the deposition of Ti_3_C_2_T_x_ nanoflakes for 30 min may overfill the 5-μm-thick CNT network. The excessively stacked nanoflakes formed a high-reflection coating. The CNT buckypaper with an excessively small thickness has a relatively low EMI SE and may be unsuitable for a long-time deposition. Thus, the 15-μm CNT buckypaper is considered to be a suitable substrate for construction of an ultrathin and high-performance EMI shielding film. Meanwhile, the significantly increased electrical conductivity of the Ti_3_C_2_T_x_@CNT hybrid buckypaper further confirms the significant contribution of the filling of Ti_3_C_2_T_x_ nanoflakes to the shielding performance (Fig. 4f). Because the pristine CNT buckypapers with different thicknesses have an approximate density, similar electrical conductivities from 7030 to 7704 S m^−1^ are presented in different CNT buckypapers. After the electrophoretic deposition process, the electrical conductivities of the hybrid films showed a distinct increase. The 100-μm hybrid buckypaper exhibits a high conductivity of 10,062 S m^−1^, which is 38.4% higher than that of the pristine CNT buckypaper. The highest conductivity of 14,933 S m^−1^ was observed in the 5-μm sample, which is 105.8% higher than that of the pristine CNT buckypaper. This also confirms that the same filling of the Ti_3_C_2_T_x_ content contributes to a higher improvement in electrical conductivity and thereby a higher EMI SE in thinner hybrid films.

**Fig. 5:**
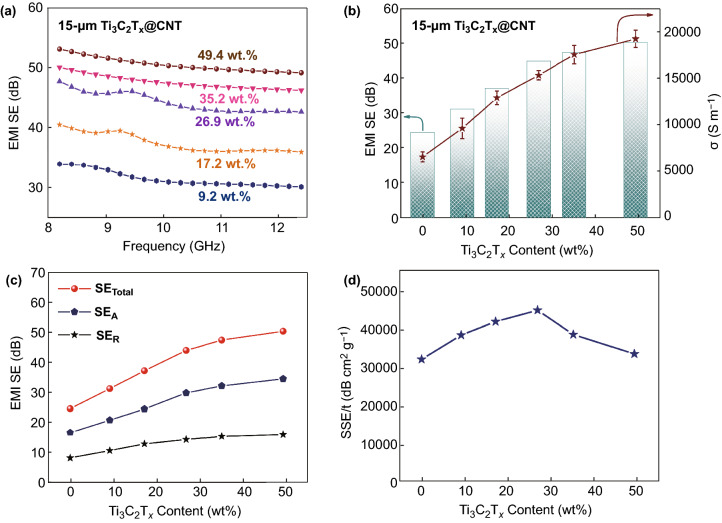
EMI performance of Ti_3_C_2_T_x_@CNT hybrid buckypapers with different Ti_3_C_2_T_x_ content. **a** EMI SE of 15-μm hybrid buckypapers with different contents of Ti_3_C_2_T_x_. **b** Comparison of average SE_Total_ and electrical conductivities versus Ti_3_C_2_T_x_ content in 15-μm hybrid buckypapers. **c** Comparison of average SE_Total_, SE_A_, and SE_R_ versus Ti_3_C_2_T_x_ content in 15-μm hybrid buckypapers. **d** SSE/t versus Ti_3_C_2_T_x_ content in 15-μm hybrid buckypapers

To construct an ultrathin and high-performance EMI shielding material, Ti_3_C_2_T_x_@CNT hybrid buckypapers with various MXene contents at a small thickness (15 μm) were further investigated. The 15-μm Ti_3_C_2_T_x_@CNT hybrid buckypapers with various Ti_3_C_2_T_x_ contents were synthesized by regulating the deposition time. Detailed EMI SE curves of the hybrid buckypapers with various Ti_3_C_2_T_x_ contents in the X-band are shown in Figs. 5a and S7a-e. The hybrid buckypapers with higher Ti_3_C_2_T_x_ contents exhibited significant improvements in EMI shielding performance. When the Ti_3_C_2_T_x_ content in the hybrid buckypaper increases from 9.2 to 49.4 wt%, the average SE_Total_ increases from 29.3 to 50.4 dB (Fig. 5b). When compared with the pristine CNT buckypaper, the hybrid buckypaper with 49.4 wt% Ti_3_C_2_T_x_ exhibited a 105% increase in the total SE. By contrast, the electrical conductivity of the hybrid buckypaper also increased with the Ti_3_C_2_T_x_ content (Fig. 5b). A high conductivity of 19,262 S m^−1^ was observed in the hybrid film with 49.4 wt% Ti_3_C_2_T_x_, which is 193% higher than that of pristine CNT buckypaper. The similar increasing trends in the average SE_Total_ and electrical conductivity demonstrate that the promotion of electrical conductivity is a major factor for the enhancement of the shielding performance. Further comparisons of SE_Total_, SE_A_, and SE_R_ are presented in Figs. 5c and S7f. Both the SE_A_ and SE_R_ in the hybrid buckypapers show a distinct increase with the added Ti_3_C_2_T_x_ content. Meanwhile, all samples with various Ti_3_C_2_T_x_ contents retained the absorption-dominant mechanism for EMI shielding.

A high EMI SE can be achieved by simply increasing the thickness. To evaluate the applied value of the EMI shielding materials, the density and thickness of the samples should be considered. The calculated thickness-averaged specific SE value (SSE/*t*), derived by dividing the SE by the density and thickness, was further investigated (Fig. 5d). Ultrahigh SSE/*t* values of over 30,000 dB cm^2^ g^−1^ are presented in all 15-μm Ti_3_C_2_T_x_@CNT hybrid buckypapers with various Ti_3_C_2_T_x_ content. Among them, the highest SSE/t value of 44,607.3 dB cm^2^ g^−1^ is observed in the sample with 26.9 wt% Ti_3_C_2_T_x_ (total density of 0.68 g cm^−3^), which is 40% higher than the value of a pristine CNT buckypaper. All of these results demonstrate that the shielding performance of Ti_3_C_2_T_x_@CNT hybrid buckypapers can be efficiently improved by increasing the Ti_3_C_2_T_x_ content. Ultrathin and high-performance Ti_3_C_2_T_x_@CNT hybrid buckypaper for EMI shielding can be effectively constructed.

To further demonstrate the superiority of the Ti_3_C_2_T_x_@CNT hybrid buckypaper developed in this work, randomly mixed Ti_3_C_2_T_x_/CNT (r-Ti_3_C_2_T_x_/CNTs) hybrid films were fabricated for comparison. The r-Ti_3_C_2_T_x_/CNT films were prepared by filtrating the mixed Ti_3_C_2_T_x_/CNT through water dispersion, in which the thicknesses are controlled in 15 μm. The EMI shielding performances of the r-Ti_3_C_2_T_x_/CNT films with different Ti_3_C_2_T_x_ contents are shown in Fig. S8a-e. This is consistent with those reported in [9, 31]. The SE of r-Ti_3_C_2_T_x_/ CNT films also shows an obvious increase with the addition of Ti_3_C_2_T_x_ content. However, the increasing trend in the r-Ti_3_C_2_T_x_/CNT film is much lower than that of the Ti_3_C_2_T_x_@CNT hybrid buckypaper (Fig. S8f). When the Ti_3_C_2_T_x_@CNT hybrid buckypaper with 49.4 wt% Ti_3_C_2_T_x_ shows a high SE of 50.4 dB, the value of an r-Ti_3_C_2_T_x_/CNT film with 60 wt% Ti_3_C_2_T_x_ is only 36.9 dB. The relatively low shielding performance of the r-Ti_3_C_2_T_x_/CNT film may be attributed to the unordered stacking structure. The overstacking of Ti_3_C_2_T_x_ nanoflakes will weaken the connection in the components, resulting in low conductivity and thus low shielding performance. In this work, through a simple electrophoretic deposition process, the Ti_3_C_2_T_x_ nanoflakes can be well dispersed in the CNT network. Each piece of nanoflake is well connected to the carbon nanotubes. The resulting continuous conductive network leads to an outstanding EMI shielding performance.

**Fig. 6:**
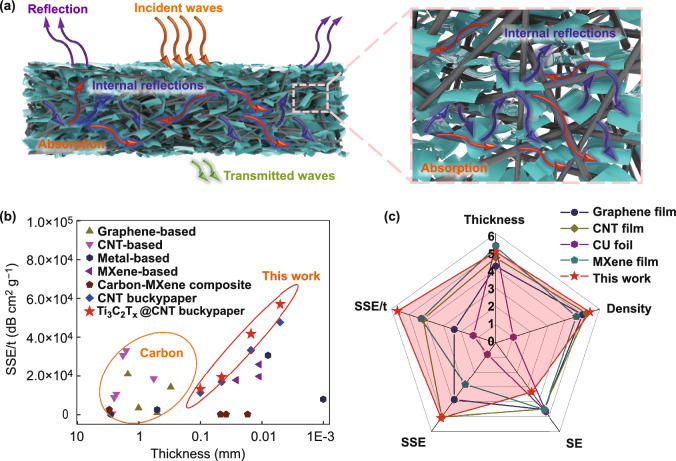
**a** Proposed EMI shielding mechanism of Ti_3_C_2_T_x_@CNT hybrid buckypaper. **b** Comparison of SSE/t versus thickness in Ti_3_C_2_T_x_@CNT hybrid buckypapers and other shielding materials. Detailed data thereof are listed in Table S1. **c** Radar chart for comparison of thickness, density, SE, SSE, and SSE/t in the optimized 15-μm Ti_3_C_2_T_x_@CNT hybrid buckypaper and the other reported ultrathin EMI shielding films with a thickness of 11–27 μm, in which the five dimensions are divided into six grades. In particular, high grades in thickness and density mean low values. Detailed data thereof are listed in Table S2

The proposed EMI shielding mechanism in the Ti_3_C_2_T_x_@CNT hybrid buckypaper is illustrated in Fig. 6a. When the electromagnetic waves reach the surface of the hybrid buckypaper, a portion of the incident waves are immediately reflected back, which is attributed to the high conductivity of the hybrid film. The remaining incident waves continuously spread into the hybrid buckypaper and interact with the high electron density of the CNT-Ti_3_C_2_T_x_ network, resulting in ohmic loss and energy dissipation. Meanwhile, the well-separated Ti_3_C_2_T_x_ nanoflakes and the formed micro-fissures in the CNT-Ti_3_C_2_T_x_ network provided an abundant air–solid interface for multiple internal reflections and interface polarization loss. The electromagnetic waves are repeatedly reflected inside the CNT-Ti_3_C_2_T_x_ network until they are completely absorbed. Moreover, the abundant defects in the slightly etched carbon nanotubes and Ti_3_C_2_T_x_ nanoflakes also lead to an asymmetric distribution of electrons and thus dielectric loss [31, 40]. All of these factors result in a high EMI shielding performance in the Ti_3_C_2_T_x_@CNT hybrid film.

To objectively evaluate the practicality, the EMI SE values and thickness-averaged SSE/t values of Ti_3_C_2_T_x_@CNT hybrid buckypapers are compared with those of other materials in Figs. 6b and S9 (the detailed data thereof are listed in Table S1). Although the reported graphene-based [6, 7, 10, 41] and CNT-based composites [4, 12, 42, 43] exhibit a high EMI SE, they all present a relatively low SSE/t owing to their high thickness. The dense metal-based film [24, 44, 45], Ti_3_C_2_T_x_ film [24], and Ti_3_C_2_T_x_/polymer film [20, 21] exhibit a high EMI SE with a small thickness. However, the high densities result in low SSE/t values. Some reported Ti_3_C_2_T_x_/carbon composites show both a high mechanical strength and shielding performance [1, 9, 28, 31]. However, their high thickness or high density still leads to a low SSE/t. The Ti_3_C_2_T_x_@CNT hybrid buckypapers developed in this work reached a balance of a relatively low density, low thickness, and high SE. All these factors contribute to the high SSE/t value (13,074.2–56,945.8 dB cm^2^ g^−1^).

A satisfactory commercial EMI shielding film requires not only a high SE, but also a small thickness, low density, and high SSE and SSE/t. The 15-μm Ti_3_C_2_T_x_@CNT hybrid buckypaper with 49.4 wt% Ti_3_C_2_T_x_ described in this work is compared with the reported ultrathin EMI shielding films (10–30 μm) such as a graphene film [46], CNT film [47], Cu foil [24], and MXene film [24] in terms of five dimensions (thickness, density, SE, SSE, and SSE/t). A radar chart, in which the five dimensions are divided into six grades (in particular, high grades in thickness and density mean low values), is shown in Fig. 6c. Detailed data thereof are shown in Table S2. The high SE in these ultrathin EMI shielding films relies on the high densities. At similar thicknesses, they all show a relatively low SSE and SSE/t. For instance, although the Cu foil shows a high EMI SE of 70 dB in a small thickness of 10 μm, its density is as high as 9.87 g cm^−3^. Thus, the Cu foil exhibits a relatively low SSE of 7.8 dB cm^3^ g^−1^ and SSE/t of 7812 dB cm^2^ g^−1^. By contrast, the 15-μm Ti_3_C_2_T_x_@CNT hybrid buckypaper with 49.4 wt% Ti_3_C_2_T_x_ in this work shows a satisfactory EMI SE of 50.4 dB with a relative low density of 0.98 g cm^−3^. Therefore, a high SSE of 51.0 dB cm^3^ g^−1^ and SSE/t of 34,003.7 dB cm^2^ g^−1^ are achieved. The high SSE and SSE/t in the hybrid buckypaper may be attributed to the well-designed internal microstructure.

## Conclusions

In this study, we report an efficient electrophoretic deposition process to construct a flexible, lightweight, and ultrathin Ti_3_C_2_T_x_@CNT hybrid buckypaper with high EMI shielding performance. The homogeneously filled Ti_3_C_2_T_x_ nanoflakes effectively improved both the reflection and absorption of the electromagnetic wave in the hybrid film. An SE_Total_ of 60.5 dB was yielded within the X-band for the 100-μm hybrid buckypaper. Meanwhile, increasing the MXene content can effectively enhance the SE. Moreover, a distinguished SSE/*t* value of 56,945.8 dB cm^2^ g^−1^ was exhibited in the 5-μm hybrid buckypaper. Thus, flexible and ultrathin Ti_3_C_2_T_x_@CNT hybrid buckypapers with an outstanding EMI shielding performance have significant applications in the fields of smart and wearable electronic devices.

## Supplementary Information

Below is the link to the electronic supplementary material.

Supplementary file1

## References

[1] Cao WT, Chen FF, Zhu YJ, Zhang YG, Jiang YY (2018). Binary strengthening and toughening of MXene/cellulose nanofiber composite paper with nacre-inspired structure and superior electromagnetic interference shielding properties. ACS Nano.

[2] Hu M, Cui C, Shi C, Wu ZS, Yang J (2019). High-energy-density hydrogen-ion-rocking-chair hybrid supercapacitors based on Ti_3_C_2_T_x_ MXene and carbon nanotubes mediated by redox active molecule. ACS Nano.

[3] Chen Y, Zhang HB, Yang Y, Wang M, Cao A (2016). High-performance epoxy nanocomposites reinforced with three-dimensional carbon nanotube sponge for electromagnetic interference shielding. Adv. Funct. Mater..

[4] Hu P, Lyu J, Fu C, Gong WB, Liao J (2020). Multifunctional aramid nanofiber/carbon nanotube hybrid aerogel films. ACS Nano.

[5] Zeng Z, Jin H, Chen M, Li W, Zhou L (2017). Microstructure design of lightweight, flexible, and high electromagnetic shielding porous multiwalled carbon nanotube/polymer composites. Small.

[6] Chen Z, Xu C, Ma C, Ren W, Cheng HM (2013). Lightweight and flexible graphene foam composites for high-performance electromagnetic interference shielding. Adv. Mater..

[7] Wu Y, Wang Z, Liu X, Shen X, Zheng Q (2017). Ultralight graphene foam/conductive polymer composites for exceptional electromagnetic interference shielding. ACS Appl. Mater. Interfaces.

[8] Weng C, Wang G, Dai Z, Pei Y, Liu L (2019). Buckled AgNW/MXene hybrid hierarchical sponges for high-performance electromagnetic interference shielding. Nanoscale.

[9] Sambyal P, Iqbal A, Hong J, Kim H, Kim MK (2019). Ultralight and mechanically robust Ti_3_C_2_T_x_ hybrid aerogel reinforced by carbon nanotubes for electromagnetic interference shielding. ACS Appl. Mater. Interfaces.

[10] Liu J, Liu Y, Zhang HB, Dai Y, Liu Z (2018). Superelastic and multifunctional graphene based aerogels by interfacial reinforcement with graphitized carbon at high temperatures. Carbon.

[11] Song Q, Ye F, Yin X, Li W, Li H (2017). Carbon nanotube–multilayered graphene edge plane core–shell hybrid foams for ultrahigh-performance electromagnetic-interference shielding. Adv. Mater..

[12] Lu D, Mo Z, Liang B, Yang L, He Z (2018). Flexible, lightweight carbon nanotube sponges and composites for high-performance electromagnetic interference shielding. Carbon.

[13] Zhan Y, Wang J, Zhang K, Li Y, Meng Y (2018). Fabrication of a flexible electromagnetic interference shielding Fe_3_O_4_@reduced graphene oxide/natural rubber composite with segregated network. Chem. Eng. J..

[14] Wu S, Zou M, Li Z, Chen D, Zhang H (2018). Robust and stable Cu nanowire@graphene core–shell aerogels for ultraeffective electromagnetic interference shielding. Small.

[15] Jung J, Lee H, Ha I, Cho H, Kim KK (2017). Highly stretchable and transparent electromagnetic interference shielding film based on silver nanowire percolation network for wearable electronics applications. ACS Appl. Mater. Interfaces.

[16] Cao MS, Cai YZ, He P, Shu JC, Cao WQ (2019). 2D MXenes: electromagnetic property for microwave absorption and electromagnetic interference shielding. Chem. Eng. J..

[17] Han M, Shuck CE, Rakhmanov R, Parchment D, Anasori B (2020). Beyond Ti_3_C_2_T_x_: MXenes for electromagnetic interference shielding. ACS Nano.

[18] Yun T, Kim H, Iqbal A, Cho YS, Lee GS (2020). Electromagnetic shielding of monolayer MXene assemblies. Adv. Mater..

[19] Chen H, Wen Y, Qi Y, Zhao Q, Qu L (2020). Pristine titanium carbide mxene films with environmentally stable conductivity and superior mechanical strength. Adv. Funct. Mater..

[20] Liu R, Miao M, Li Y, Zhang J, Cao S (2018). Ultrathin biomimetic polymeric Ti_3_C_2_T_x_ MXene composite films for electromagnetic interference shielding. ACS Appl. Mater. Interfaces.

[21] Zhou Z, Liu J, Zhang X, Tian D, Zhan Z (2019). Ultrathin MXene/calcium alginate aerogel film for high-performance electromagnetic interference shielding. Adv. Mater. Interfaces.

[22] Wan YJ, Li XM, Zhu PL, Sun R, Wong CP (2020). Lightweight, flexible MXene/polymer film with simultaneously excellent mechanical property and high-performance electromagnetic interference shielding. Compos. Part A.

[23] Jin X, Wang J, Dai L, Liu X, Li L (2020). Flame-retardant poly(vinyl alcohol)/mxene multilayered films with outstanding electromagnetic interference shielding and thermal conductive performances. Chem. Eng. J..

[24] Shahzad F, Alhabeb M, Hatter CB, Anasori B, Hong SM (2016). Electromagnetic interference shielding with 2D transition metal carbides (MXenes). Science.

[25] Iqbal A, Shahzad F, Hantanasirisakul K, Kim MK, Kwon J (2020). Anomalous absorption of electromagnetic waves by 2D transition metal carbonitride Ti_3_CNT_x_(MXene). Science.

[26] Lipton J, Weng GM, Rӧhr JA, Wang H, Taylor AD (2020). Layer-by-layer assembly of two-dimensional materials: meticulous control on the nanoscale. Matter.

[27] Zang X, Wang J, Qin Y, Wang T, He C (2020). Enhancing capacitance performance of ­MXene as electrode materials of supercapacitor: from controlled preparation to composite structure construction. Nano-Micro Lett..

[28] Xie F, Jia F, Zhuo L, Lu Z, Si L (2019). Ultrathin MXene/aramid nanofiber composite paper with excellent mechanical properties for efficient electromagnetic interference shielding. Nanoscale.

[29] Li Y, Tian X, Gao SP, Jing L, Li K (2020). Reversible crumpling of 2D titanium carbide (MXene) nanocoatings for stretchable electromagnetic shielding and wearable wireless communication. Adv. Funct. Mater..

[30] Zeng Z, Wang C, Siqueira G, Han D, Huch A (2020). Nanocellulose-MXene biomimetic aerogels with orientation-tunable electromagnetic interference shielding performance. Adv. Sci..

[31] Cao W, Ma C, Tan S, Ma M, Wan P (2019). Ultrathin and flexible CNTs/MXene/cellulose nanofibrils composite paper for electromagnetic interference shielding. Nano-Micro Lett..

[32] Liu J, Liu Z, Zhang HB, Chen W, Zhao Z (2020). Ultrastrong and highly conductive MXene-based films for high-performance electromagnetic interference shielding. Adv. Electron. Mater..

[33] Weng GM, Li J, Alhabeb M, Karpovich C, Wang H (2018). Layer-by-layer assembly of cross-functional semi-transparent MXene-carbon nanotubes composite films for next-generation electromagnetic interference shielding. Adv. Funct. Mater..

[34] Zhou B, Zhang Z, Li Y, Han G, Feng Y (2020). Robust, and multifunctional electromagnetic interference shielding film with alternating cellulose nanofiber and MXene layers. ACS Appl. Mater. Interfaces.

[35] Ma Z, Kang S, Ma J, Shao L, Zhang Y (2020). Ultraflexible and mechanically strong double-layered aramid nanofiber–Ti_3_C_2_T_x_ MXene/silver nanowire nanocomposite papers for high-performance electromagnetic interference shielding. ACS Nano.

[36] Cano-Márquez AG, Rodríguez-Macías FJ, Campos-Delgado J, Espinosa-González CG, Tristán-López F (2009). Ex-MWNTs: graphene sheets and ribbons produced by lithium intercalation and exfoliation of carbon nanotubes. Nano Lett..

[37] Lim J, Maiti UN, Kim NY, Narayan R, Lee WJ (2016). Dopant-specific unzipping of carbon nanotubes for intact crystalline graphene nanostructures. Nat. Commun..

[38] Yang S, Zhang P, Wang F, Ricciardulli AG, Lohe MR (2018). fluoride-free synthesis of two-dimensional titanium carbide (MXene) using a binary aqueous system. Angew. Chem. Int. Ed..

[39] Schultz T, Frey NC, Hantanasirisakul K, Park S, May SJ (2019). Surface termination dependent work function and electronic properties of Ti_3_C_2_T_x_ MXene. Chem. Mater..

[40] Cui C, Xiang C, Geng L, Lai X, Guo R (2019). Flexible and ultrathin electrospun regenerate cellulose nano fibers and D-Ti_3_C_2_T_x_ ( MXene ) composite film for electromagnetic interference shielding. J. Alloys Compd..

[41] Shen B, Li Y, Yi D, Zhai W, Wei X (2016). Microcellular graphene foam for improved broadband electromagnetic interference shielding. Carbon.

[42] Tan YJ, Li J, Cai JH, Tang XH, Liu JH (2019). Comparative study on solid and hollow glass microspheres for enhanced electromagnetic interference shielding in polydimethylsiloxane/multi-walled carbon nanotube composites. Compos. Part B.

[43] Li M, Jia L, Zhang X, Yan D, Zhang Q (2018). Robust carbon nanotube foam for efficient electromagnetic interference shielding and microwave absorption. J. Colloid Interface Sci..

[44] Shui X, Chung DDL (1997). Nickel filament polymer–matrix composites with low surface impedance and high electromagnetic interference shielding effectiveness. J. Electron. Mater..

[45] Ma J, Wang K, Zhan M (2015). A comparative study of structure and electromagnetic interference shielding performance for silver nanostructure hybrid polyimide foams. RSC Adv..

[46] Zhou E, Xi J, Liu Y, Xu Z, Guo Y (2017). Large-area potassium-doped highly conductive graphene films for electromagnetic interference shielding. Nanoscale.

[47] Li H, Lu X, Yuan D, Sun J, Erden F (2017). Lightweight flexible carbon nanotube/polyaniline films with outstanding EMI shielding properties. J. Mater. Chem. C.

