# Supplementary material for: Ultrathin, Lightweight, and Flexible CNT Buckypaper Enhanced Using MXenes for Electromagnetic Interference Shielding
Source: Nanomicro Lett. 2021 Feb 9;13:66. doi: 10.1007/s40820-021-00597-4 (PMC8187523; doi:10.1007/s40820-021-00597-4)
Supplement: Supplementary file 1 — Supplementary file1 [file 40820_2021_597_MOESM1_ESM.pdf]

Supporting Information for

Ultrathin, Lightweight, and Flexible CNT Buckypaper Enhanced Using MXenes for Electromagnetic Interference Shielding

Rongliang Yang[1], Xuchun Gui[1,*], Li Yao[2], Qingmei Hu[1], Leilei Yang[1], Hao Zhang[3], Yongtao Yao[4], Hui Mei[2,*], Zikang Tang[5]

[1]State Key Laboratory of Optoelectronic Materials and Technologies, School of Electronics and Information Technology, Sun Yat-sen University, Guangzhou 510275, P. R. China

[2]Science and Technology on Thermostructural Composite Materials Laboratory, School of Materials Science and Engineering, Northwestern Polytechnical University, Xi'an, Shaanxi 710072, P. R. China

[3]Instrumental Analysis and Research Center (IARC), Sun Yat-sen University, Guangzhou 510275, P. R. China

[4]National Key Laboratory of Science and Technology on Advanced Composites in Special Environments, Harbin Institute of Technology, Harbin 150080, P. R. China

[5]Institute of Applied Physics and Materials Engineering, University of Macau, Taipa 999078, Macau, P. R. China

*Corresponding authors. E-mail: guixch@mail.sysu.edu.cn (Xuchun Gui); phdhuimei@yahoo.com (Hui Mei)

Supplementary Tables and Figures

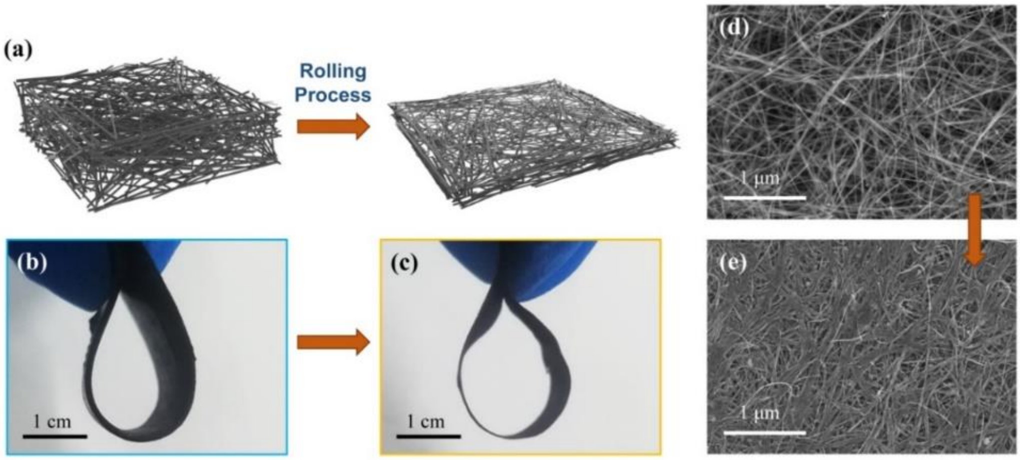

Fig. S1 a Schematic illustration of rolling process for densifying the CNT buckypaper. Digital image of CNT buckypaper **b** before and **c** after densifying. Top-view SEM image of CNT buckypaper **d** before and **e** after densifying

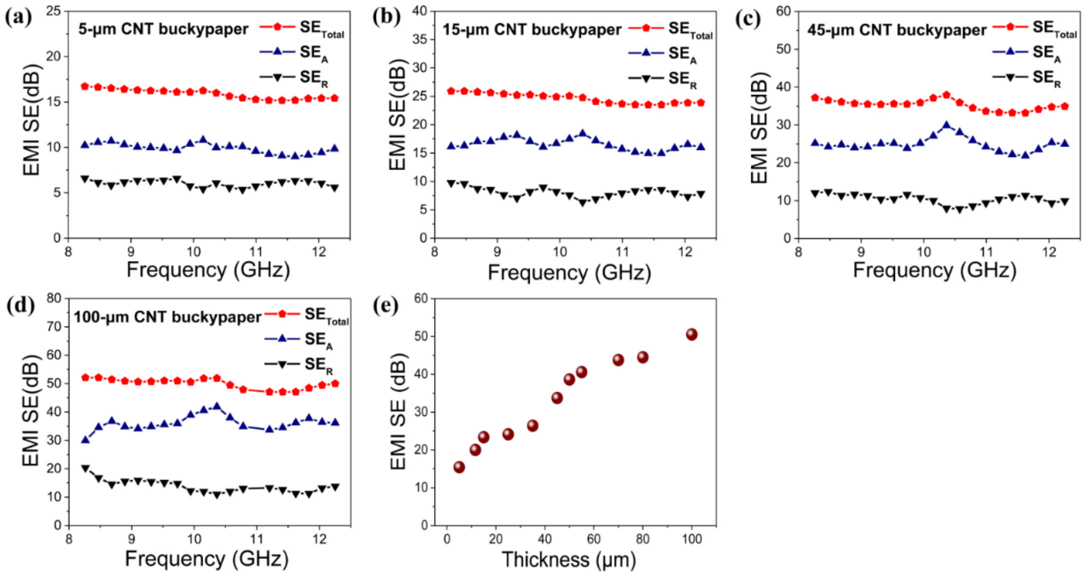

Fig. S2 EMI SE of CNT buckypapers with thicknesses of **a** 5 μm, **b** 15 μm, **c** 45 μm, and **d** 100 μm in X-band region. **e** Comprehensive average SE_{Total} data *versus* thickness of CNT buckypapers

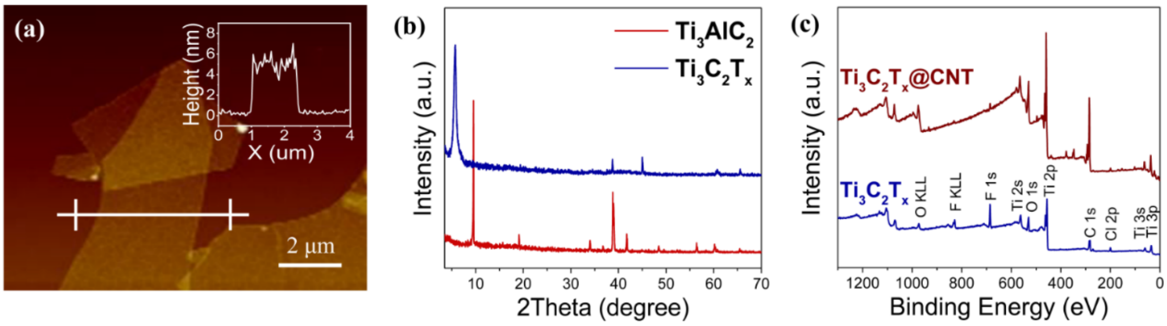

Fig. S3 a AFM image of $Ti_3C_2T_x$ nanosheets. **b** XRD patterns of Ti_3AlC_2 powder and $Ti_3C_2T_x$ nanosheets. **c** XPS survey spectra for $Ti_3C_2T_x$ and $Ti_3C_2T_x$@CNT hybrid buckypaper

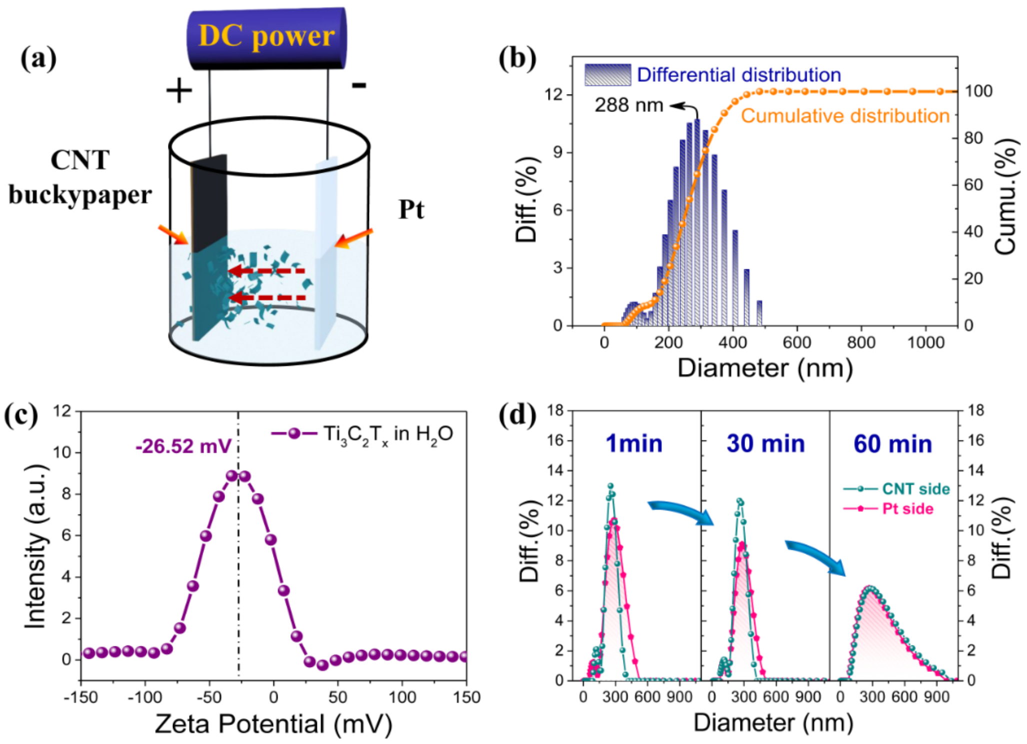

Fig. S4 a Schematic illustration of electrophoretic deposition process. **b** Size distribution and **c** zeta potential of $Ti_3C_2T_x$ nanoflakes in the $Ti_3C_2T_x$ aqueous dispersion. **d** Variation of size distributions in $Ti_3C_2T_x$ nanoflakes around two electrodes during the electrophoretic deposition process

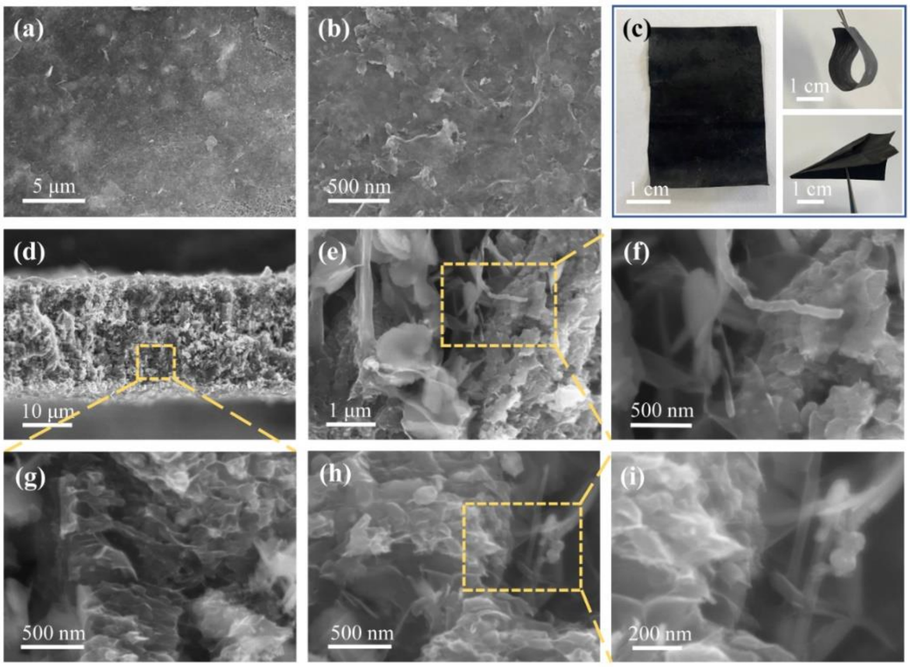

Fig. S5 a, b Top-view SEM images of $Ti_3C_2T_x$@CNT hybrid buckypaper. **c** Digital images of $Ti_3C_2T_x$@CNT hybrid buckypapers. **d-i** Cross-sectional SEM images of $Ti_3C_2T_x$@CNT hybrid buckypaper

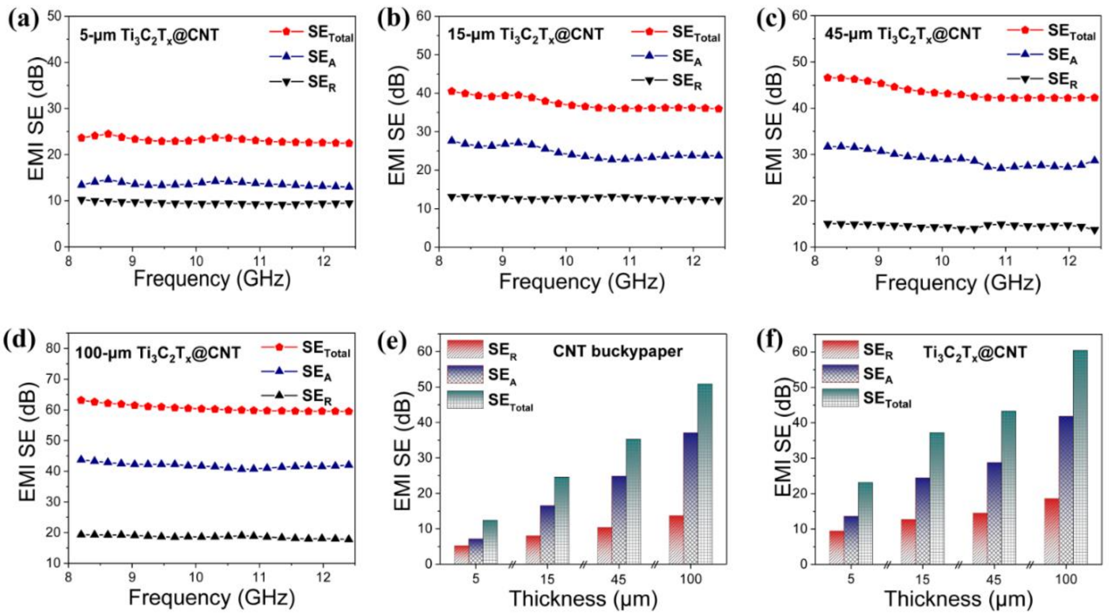

Fig. S6 EMI SE of $Ti_3C_2T_x$@CNT hybrid buckypapers with thicknesses of **a** 5 μm, **b** 15 μm, **c** 45 μm, and **d** 100 μm in X-band region. Comparison of average SE_R, SE_A, and SE_{Total} versus thickness in **e** CNT buckypapers and **f** $Ti_3C_2T_x$@CNT hybrid buckypapers

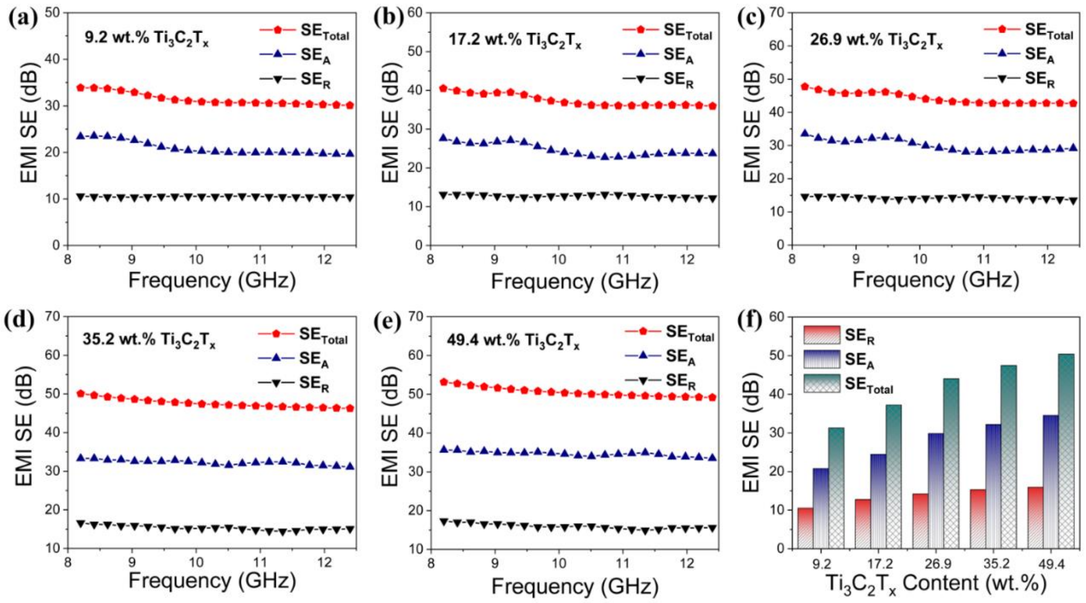

Fig. S7 EMI SE of $Ti_3C_2T_x$ @CNT hybrid buckypapers with $Ti_3C_2T_x$ content of **a** 9.2 wt%, **b** 17.2 wt%, **c** 26.9 wt%, **d** 35.2 wt%, and **e** 49.4 wt%. **f** Comparison of average SE_R, SE_A, and SE_{Total} versus $Ti_3C_2T_x$ content in $Ti_3C_2T_x$@CNT hybrid buckypapers

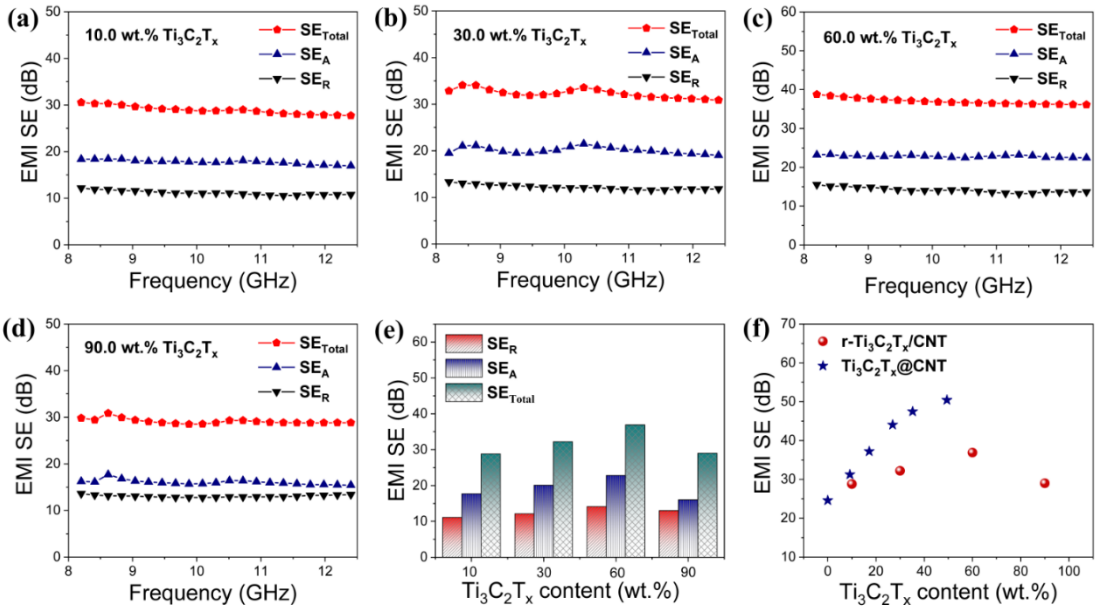

Fig. S8 EMI SE of randomly mixed $Ti_3C_2T_x$ /CNT films with $Ti_3C_2T_x$ content of **a** 10.0 wt%, **b** 30.0 wt%, **c** 60.0 wt%, and **d** 90.0 wt% in X-band region. **e** Comparison of average SE_R, SE_A, and SE_{Total} versus $Ti_3C_2T_x$ content in r-$Ti_3C_2T_x$ /CNT films. **f** Comparison of average SE_{Total} versus $Ti_3C_2T_x$ content between $Ti_3C_2T_x$@CNT hybrid buckypapers and r-$Ti_3C_2T_x$ /CNT films

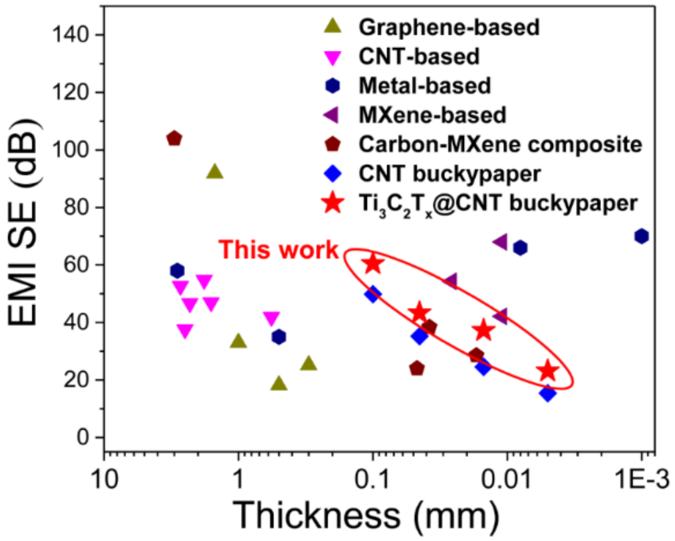

Fig. S9 Comparison of SE versus thickness in $Ti_3C_2T_x$@CNT hybrid buckypapers and other shielding materials. Detailed data thereof is listed in Table S1

Table S1 EMI shielding performance, thickness averaged specific EMI SE of various shielding materials

Type	Materials	Thickness (mm)	SE (dB)	SSE/t (dB cm^2 g^{-1})	Refs.
Graphene-based	Graphene foam/PDMS	1	33	3330	[S1]
	Graphene foam/ PEDOT/PSS	1.5	91.9	20800	[S2]
	RGO/MWCNTs/PI	0.5	18.2	823	[S3]
	Graphene foam	0.3	25.2	14000	[S4]
CNT-based	CNT/WPU	2.3	46.7	10400	[S5]
	MWCNT/PDMS/ hollow glass microspheres	2.7	52.7	260.4	[S6]
	CNT/Aramid Nanofiber	0.568	41.9	18304.6	[S7]
	CNT/Graphene Edge hybrid foam	1.6	47	33005.6	[S8]
	CNT/Chitosan	2.5	37.6	8556	[S9]
	CNT sponge	1.8	54.8	30444	[S10]
Metal-based	Ni fiber/PES	2.85	58	109	[S11]
	Ag NW	0.5	35	2416	[S12]
	Al Foil	0.008	66	30555	[S13]
	Cu Foil	0.001	70	7812	
MXene-based	$Ti_3C_2T_x$	0.011	68	25863	[S13]
	$Ti_3C_2T_x$ /CA	0.026	54.3	17586	[S14]
	$Ti_3C_2T_x$ /PEDOT/PSS	0.011	42.1	19497.8	[S15]
Carbon-MXene composite	Aramid nanofiber/ $Ti_3C_2T_x$	0.017	28.5	13377.1	[S16]
	Cellulose Nanofiber/ $Ti_3C_2T_x$	0.047	24	2647	[S17]
	CNTs/ $Ti_3C_2T_x$ / Cellulose Nanofibrils	0.038	38.4	8020	[S18]
	CNTs/ $Ti_3C_2T_x$ aerogel	3	104	8253.2	[S19]
This work	CNT buckypapers	0.1	49.8	11127.3	This work
		0.045	35.2	16721.7	
		0.015	24.6	33182.8	
		0.005	15.4	47512.8	
This work	$Ti_3C_2T_x$@CNT hybrid buckypaper	0.1	60.5	13074.2	This work
		0.045	43.3	19138.4	
		0.015	37.2	41635.7	
		0.005	23.1	56945.8	

Table S2 Thickness, density, SE, SSE, SSE/t of various ultrathin EMI shielding films

Materials	Thickness (μm)	Density (g cm^{-3})	SE (dB)	SSE (dB cm^3 g^{-1})	SSE/t (dB cm^2 g^{-1})	Refs.
Graphene film	27	1.76	68	38.6	14309.8	[S20]
CNT film	20	1.34	67.4	50.3	25149.3	[S21]
Cu foil	10	8.97	70	7.8	7812	[S13]
MXene film	11	2.39	68	28.4	25863	[S13]
$Ti_3C_2T_x$@CNT buckypaper	15	0.98	50.4	51.0	34003.7	This work

Supplementary References

[S1] Z. Chen, C. Xu, C. Ma, W. Ren, H. M. Cheng, Lightweight and flexible graphene foam composites for high-performance electromagnetic interference shielding. Adv. Mater. **25**, 1296 (2013). https://doi.org/10.1002/adma.201204196

[S2] Y. Wu, Z. Wang, X. Liu, X. Shen, Q. Zheng et al., Ultralight graphene foam/conductive polymer composites for exceptional electromagnetic interference shielding. ACS Appl. Mater. Interfaces **9**, 9059 (2017). https://doi.org/10.1021/acsami.7b01017

[S3] H. Yang, Z. Yu, P. Wu, H. Zou, P. Liu, Electromagnetic interference shielding effectiveness of microcellular polyimide / in situ thermally reduced graphene oxide / carbon nanotubes nanocomposites. Appl. Surf. Sci. **434**, 318 (2018). https://doi.org/10.1016/j.apsusc.2017.10.191

[S4] B. Shen, Y. Li, D. Yi, W. Zhai, X. Wei et al., Microcellular graphene foam for improved broadband electromagnetic interference shielding. Carbon **102**, 154 (2016). https://doi.org/10.1016/j.carbon.2016.02.040

[S5] Z. Zeng, H. Jin, M. Chen, W. Li, L. Zhou et al., Microstructure design of lightweight, flexible, and high electromagnetic shielding porous multiwalled carbon nanotube/polymer composites. Small **13**, 1 (2017). https://doi.org/10.1002/smll.201701388

[S6] Y. J. Tan, J. Li, J. H. Cai, X. H. Tang, J. H. Liu et al., Comparative study on solid and hollow glass microspheres for enhanced electromagnetic interference shielding in polydimethylsiloxane/multi-walled carbon nanotube composites. Compos. Part B **177**, 107378 (2019). https://doi.org/10.1016/j.compositesb.2019.107378

[S7] P. Hu, J. Lyu, C. Fu, W. Bin Gong, J. Liao et al., Multifunctional aramid nanofiber/carbon nanotube hybrid aerogel films. ACS Nano **14**, 688 (2020). https://doi.org/10.1021/acsnano.9b07459

[S8] Q. Song, F. Ye, X. Yin, W. Li, H. Li, Y. Liu, K. Li, K. Xie, X. Li, Q. Fu, L. Cheng, L. Zhang, B. Wei, et al., Carbon nanotube–multilayered graphene edge plane core–shell hybrid foams for ultrahigh-performance electromagnetic-interference shielding. Adv. Mater. **29**, 1 (2017). https://doi.org/10.1002/adma.201701583

[S9] M. Li, L. Jia, X. Zhang, D. Yan, Q. Zhang et al., Robust carbon nanotube foam for efficient electromagnetic interference shielding and microwave absorption. J. Colloid Interface Sci. **530**, 113 (2018). https://doi.org/10.1016/j.jcis.2018.06.052

[S10] D. Lu, Z. Mo, B. Liang, L. Yang, Z. He et al., Flexible , Lightweight carbon nanotube sponges and composites for high-performance electromagnetic interference shielding. Carbon **133**, 457 (2018). https://doi.org/10.1016/j.carbon.2018.03.061

[S11] X. Shui, D. D. L. Chung, Nickel filament polymer-matrix composites with low surface impedance and high electromagnetic interference shielding effectiveness. J. Electron. Mater. **26**, 928 (1997). https://doi.org/10.1007/s11664-997-0276-4

[S12] J. Ma, K. Wang, M. Zhan, A comparative study of structure and ehlectromagnetic interference shielding performance for silver nanostructure hybrid polyimide foams. RSC Adv. **5**, 65283 (2015). https://doi.org/10.1039/c5ra09507g

[S13] F. Shahzad, M. Alhabeb, C. B. Hatter, B. Anasori, S. M. Hong et al., Electromagnetic interference shielding with 2D transition metal carbides (MXenes). Science **353**, 1137 (2016). https://doi.org/10.1126/science.aag2421

[S14] Z. Zhou, J. Liu, X. Zhang, D. Tian, Z. Zhan et al., Ultrathin MXene/calcium alginate aerogel film for high-performance electromagnetic interference shielding. Adv. Mater. Interfaces **6**, 1 (2019). https://doi.org/10.1002/admi.201802040

[S15] R. Liu, M. Miao, Y. Li, J. Zhang, S. Cao et al., Ultrathin biomimetic polymeric $Ti_3C_2T_x$ MXene composite films for electromagnetic interference shielding. ACS Appl. Mater. Interfaces **10**, 44787 (2018). https://doi.org/10.1021/acsami.8b18347

[S16] F. Xie, F. Jia, L. Zhuo, Z. Lu, L. Si et al., Ultrathin MXene/aramid nanofiber composite paper with excellent mechanical properties for efficient electromagnetic interference shielding. Nanoscale **11**, 23382 (2019). https://doi.org/10.1039/c9nr07331k

[S17] W. T. Cao, F. F. Chen, Y. J. Zhu, Y. G. Zhang, Y. Y. Jiang et al., Binary strengthening and toughening of MXene/cellulose nanofiber composite paper with nacre-inspired structure and superior electromagnetic interference shielding properties. ACS Nano **12**, 4583 (2018). https://doi.org/10.1021/acsnano.8b00997

[S18] W. Cao, C. Ma, S. Tan, M. Ma, P. Wan et al., Ultrathin and flexible CNTs/MXene/cellulose nanofibrils composite paper for electromagnetic interference shielding. Nano-Micro Lett. **11**, 1 (2019). https://doi.org/10.1007/s40820-019-0304-y

[S19] P. Sambyal, A. Iqbal, J. Hong, H. Kim, M. K. Kim et al.,Ultralight and mechanically robust $Ti_3C_2T_x$ hybrid aerogel reinforced by carbon nanotubes for electromagnetic interference shielding. ACS Appl. Mater. Interfaces **11**, 38046 (2019). https://doi.org/10.1021/acsami.9b12550

[S20] E. Zhou, J. Xi, Y. Liu, Z. Xu, Y. Guo, et al., Large-area potassium-doped highly conductive graphene films for electromagnetic interference shielding. Nanoscale **9**, 18613 (2017). https://doi.org/10.1039/c7nr07030f

[S21] H. Li, X. Lu, D. Yuan, J. Sun, F. Erden et al., Lightweight flexible carbon nanotube/polyaniline films with outstanding EMI shielding properties. J. Mater. Chem. C **5**, 8694 (2017). https://doi.org/10.1039/c7tc02394d
